# In this issue of Current Oncology

**Published:** 2009-03

**Authors:** M. McLean

“Privatization is not an answer to health care access problems, increased public funding is,” or so claims Ervin B. Podgorsak in the first of our guest editorials in this issue of *Current Oncology.* To rescue a Canadian health care system that is of high quality, but that has problems with access and cost, the author suggests an immediate cash bailout of between 15% and 20% of funding to meet stated goals—a solution possibly difficult to envisage in these economic times, no matter the eloquence of the argument.

“Privately run health care ... results in a U.S.-type two-tiered and socially unjust medical system in which access to health care depends on patients’ ability to pay for services rather than on the need for them.” This is a pivotal argument in the manuscript and one that I have always had some difficulty with. If someone elects to pay for their arthroplasty in a private facility, then presumably one less individual is waitlisted elsewhere. A win–win.

Is it “fair” to allow this situation to happen? I don’t know the answer. Is it fair to force someone to wait for services that they can afford to purchase in the private domain, where it available? Perhaps we should attempt to improve the efficiency of the system, eliminate the appalling cost of waste and duplication, adhere strictly to a policy of evidence-based medicine, inject funds (if any are left after the banking fiascos), and expand private practice.

Also in this issue, a second guest editorial from Dr. Patricia Dobkin considers how a physician’s mindfulness may facilitate healing in the context of medical practice, and Dr. O. Graciela Scharovsky and her colleagues discuss the concept of metronomic chemotherapy. This latter approach of chronic, equally spaced administration of generally low doses of various chemotherapeutic drugs without extended rest periods departs from the more conventional pulsed administration of a maximal tolerated dose. The metronomic dosing concept is explored, and its possible advantages are discussed.

In the Radiation Oncology section, measurement of quality of life in cancer patients receiving palliative radiotherapy for symptomatic lung cancer is discussed. This important role for radiotherapy departments is expanding with the inception of rapid-response programs serving the community. (A paper in issue 3 of the current volume will discuss treatment delivery by image-guided intensity-modulated radiotherapy for this group of patients, a technique hitherto typically reserved for more radical and curative treatment options.)

In the new Psychosocial Oncology section, Drs. Robert Rutledge and Lynne Robinson argue for greater integration of hospital-based cancer care with established community services and organizations. Meanwhile, two of the journal’s regular features look at new and intriguing oncologic treatment possibilities. Updates and Developments in Oncology presents a discussion of electroporation-based cancer treatment approaches that are currently undergoing intensive investigation in the fields of drug delivery and gene therapy for the electrochemotherapy of tumours. This technique seeks to enhance the transportation of chemotherapeutic drugs across the cell membrane to improve the therapeutic ratio. And in the Drug Development in Contemporary Oncology section, the potential role of inhibitors of the cyclin-dependent kinases (cdks), a new class of anticancer agents, is explored specifically, the use of these agents in combination with traditional cytotoxic chemotherapy. In combination, these new agents appear to have potential to assist in overcoming drug resistance, thereby improving cytotoxic efficacy.

